# Effect of a structured Maintenance of Certification-based training program on perceived stress among nursing students preparing for Egypt’s new pre-licensure examination: A quasi-experimental study

**DOI:** 10.1097/MD.0000000000050037

**Published:** 2026-07-31

**Authors:** Hassan Ahmed Awad Basyouny, Mohamed Hashem Kotp, Abdelaziz Hendy, Rasha Kadri Ibrahim, Hossam Ali Ismail, Ahmed Shaaban Attia, Ahmed Khalaf Mekdad, Ahmed Ali Hafez, Eman Atef Ali, Rasha Mohammed Hussien, Shimaa Ramadan Ahmed, Wafa Hamad Almegewly, Sameer A Alkubati, Mohamed Ahmed Aly

**Affiliations:** a Nursing Administration, Faculty of Nursing, Helwan University, Cairo, Egypt; b Department of Nursing, College of Applied Medical Sciences, King Faisal University, Al Ahsa, Saudi Arabia; c Nursing Administration, Department of Nursing, Fakeeh College for Medical Sciences, Jeddah, Saudi Arabia; d Pediatric Nursing, Faculty of Nursing, Ain Shams University, Cairo, Egypt; e Nursing Department, Fatima College of Health Sciences, Madinat Zayed, Al Dhafra Region, UAE; f Critical Care Nursing, Faculty of Nursing, Helwan University, Cairo, Egypt; g Specialized Health Unit, Applied College, King Faisal University, Al Ahsa, Saudi Arabia; h Clinical Nursing Sciences Department, Al Rayan National College of Nursing, Al Rayan National Colleges, Al-Madinah Al-Munawarah, Saudi Arabia; i College of Nursing, Northern Border University, Arar, Saudi Arabia; j Obstetrics and Gynecology Nursing Department, Faculty of Nursing, Helwan University, Cairo, Egypt; k Department of Community, Psychiatric and Mental Health Nursing, College of Nursing, Qassim University, Buraydah, Saudi Arabia; l Critical Care and Emergency Nursing Department, Faculty of Nursing, Beni-Suef University, Beni-Suef, Egypt; m Department of Community and Psychiatric Mental Health Nursing, College of Nursing, Princess Nourah bint Abdulrahman University, Riyadh, Saudi Arabia; n Department of Medical Surgical Nursing, College of Nursing, University of Ha’il, Ha’il City, Saudi Arabia; o Nursing Administration, Prince Sattam Bin Abdulaziz University, Wadi ad-Dawasir, Saudi Arabia.

**Keywords:** licensure examination, Maintenance of Certification, nursing education, professional competence, quasi-experimental study, stress

## Abstract

The introduction of Egypt’s national nursing pre-licensure examination has created new academic and psychological challenges for final-year nursing students, particularly because of limited familiarity with the examination format and insufficient structured preparation resources. Although combined examination-preparation and stress-management programs may improve students’ readiness, evidence regarding their effectiveness in reducing perceived stress among Egyptian nursing students remains limited. To evaluate the effect of a structured Maintenance of Certification-based training program on perceived stress among final-year nursing students preparing for the Egyptian national pre-licensure examination. A quasi-experimental comparative study was conducted among 1000 final-year nursing students recruited from 8 nursing faculties across Egypt. Participants were allocated to an intervention group that received a four-week structured training program comprising theoretical review, mock examinations, individualized feedback, and stress-management strategies, or a control group that continued with the standard curriculum. Perceived stress was assessed before and after the intervention using the 10-item Perceived Stress Scale. Data were analyzed using independent-samples and paired-samples *t*-tests, analysis of variance, and multiple linear regression. Baseline perceived stress scores were comparable between the intervention and control groups (28.8 ± 5.7 vs 29.2 ± 6.0, *P* > .05). Following the intervention, the intervention group had a significantly lower mean stress score than the control group (12.1 ± 3.2 vs 31.1 ± 7.1, *P* < .001). Perceived stress decreased significantly within the intervention group, whereas it increased significantly within the control group (both *P* < .001). Higher academic GPA, good computer skills, and prior knowledge of the pre-licensure examination were significant predictors of lower post-intervention stress, while smoking was associated with higher stress. The regression model explained 54% of the variance in post-intervention perceived stress. The structured training program was associated with a substantial reduction in perceived stress among nursing students preparing for Egypt’s national pre-licensure examination. Integrating structured examination preparation and psychological support into final-year nursing curricula may improve students’ readiness for the transition from education to professional practice.

## 1. Introduction

The pre-licensure exam for nurses is an essential global method to evaluate the readiness of nursing graduates to undertake safe and effective nursing practice.^[1,2]^ It identifies nursing graduates’ knowledge, skills, and abilities to undertake professional practice. Consequently, regulation and accreditation of pre-licensure nursing programs are committed to quality assurance and to the prevention of harm to the public.^[3,4]^ Worldwide, Various certifying organizations, such as the American Nurses Credentialing Center,^[5]^ the American Association of Critical-Care Nurses and the National Certification Corporation,^[6]^ offer certification renewal programs.^[7–9]^

Maintenance of Certification (MOC) is an ongoing educational framework following the initial grant of certification. MOC focuses on the continued inclusion of competency assessment and self-appraisal to incorporate new information to practice and evaluate performance. Designed to support the sustained professional competence, quality, and safety of health care practice, these processes are utilized regularly by practice nurses.^[9,10]^ Although practice nurses frequently engage with MOC frameworks, there is little research on the use of MOC frameworks by nursing students preparing for licensure, especially in newly developed national systems of professional licensure.

Recently, Egypt has implemented a standardized pre-licensure exam for nurses as part of reforms aimed at advancing nursing education and better meeting international standards. A bachelor’s degree in nursing in Egypt takes 5 years, as per the Ministry of Higher Education and Scientific Research Regulation No. 49 of 1972. The Egyptian Health Council Law No. 12 of 2022, issued in March 2022, established the Egyptian Health Council. To practice as a nurse in Egypt, one has to pass the national nursing licensure examination administered by the Egyptian Health Council. The initial license to practice is for 5 years and can be renewed for an additional 5 years.^[11,12]^

The National Nursing Licensure (NLE) represents a significant achievement in regulatory reform, as it establishes professional accountability and a uniform standard of competency for graduates, and ultimately assures the public that nursing care practice is safe and of high quality. The effects of the NLE, however, have created important challenges from an educational and an organizational perspective. The national nursing licensure examination has created new educational placement standards and regulatory processes for students and schools of nursing. Many students experienced anxiety, attributed to the lack of available preparatory resources, limited institutional support for understanding the examination requirements and regulations, and a general uncertainty regarding the examination expectations.^[13]^ The challenges of implementing the national nursing licensure examination extend beyond the examination itself and inhibit students’ psychological preparation to practice nursing.

Evidence from countries that have adopted national licensure examinations and studies targeting early implementation suggest that examination clarity, preparation and institutional readiness, and validity of licensure examinations are typical challenges in the early implementation stages. They adversely impact student performance, preparedness and self-efficacy.^[14]^ Moreover, the successful enforcement of licensure policies must address students as well as the availability and readiness of adequate organizations, systems, and educational infrastructures. Absence of these implementation blocks can create stress and impact the confidence of students in the examination process.^[15]^ This means that the degree of stress students experience with the implementation of national licensure examinations mostly hinges on the state of the implementations.

Nursing students face a multitude of stressors during their transition to pre-licensure exams, which can significantly impact their mental health and academic performance.^[16]^ Understanding these primary sources of stress is crucial for developing effective support systems to aid students in managing their stress levels and succeeding in their exams. They experience significant stress from high-stakes exams that are critical to their progression and eventual licensure, as well as from unfamiliar assessment formats, unclear expectations, limited orientation, and inadequate preparation. This stress is often exacerbated by the pressure to perform well, as poor performance can have severe academic and career implications.^[17]^

Clinical placements are a significant source of stress for nursing students. The fear of making mistakes, handling technical equipment, and dealing with unknown situations in a real-world healthcare setting can be overwhelming,^[18]^ in addition to disparities between theory and practice, conflicting expectations, insufficient briefings, and an overwhelming workload.^[19]^ Although students gain opportunities to practice during their clinical placements, the practice sessions are not always structured or supervised by an expert registered nurse, highlighting the need for structured repeat revision programs in the final year of the course to support students in managing anxiety and improving readiness for the licensure examination.^[20]^

From a comprehensive psychological and psychiatric perspective, alexithymia, along with emotional dysregulation and anxiety vulnerability, is increasingly recognized as a group of transdiagnostic processes that illustrate how people interpret and react to stress. Defined as a difficulty in understanding and verbalizing one’s feelings, combined with an inability to distinguish emotions from physiological sensations and an emphasis on external events, alexithymia has been associated with anxiety disorders, broader psychiatric morbidity and lesser functional capability, as well as a diminished therapeutic response.^[21,22]^

Emotional dysregulation is seen as a transdiagnostic vulnerability for internalizing and externalizing psychopathologies. In this view, difficulties with top-down regulation and negatively valenced emotionality are seen to create pathways to psychological distress and potential psychopathology.^[23]^ Related to this, models of anxiety suggest that response to stress encompasses emotional and biological sources of vulnerability, a sense of uncontrollability, and cognitive distortions that promote a focus on negative cues and threat perception of a largely uncertain nature.^[24]^

Within this context, the deficit of emotional processing may explain the variation in the outcome of stress control methods, due to the presence of problems in emotional awareness, emotional labeling, and regulation which can obstruct the recognition of stress, the selection of an appropriate coping response, and the full participation in the effortful psychological activities required by the methods.^[22,25]^

Consequently, deficits in emotional processing may be considered as factors that moderate the effects of stress management training. Some students may not improve as much as others after the implementation of traditional approaches to the management of stress because they have poor emotional self-monitoring as a result of poor stress recognition and regulation of emotions. Therefore, stress management programs that are based on the principles of emotional awareness and regulation will be of greater benefit to students who exhibit higher levels of emotional processing.^[26]^

While MOC-based training for nursing students has clear advantages, few studies have examined its effects on students preparing for licensure in Egypt. The majority of current studies focus on the effects of such training on practicing nurses in high-income countries, creating a considerable gap in understanding its effects on pre-licensure nurses in low- and middle-income countries where educational reforms are implemented. The effects of MOC-based training on stress management and exam preparedness should be researched to identify strategies that enhance students’ preparedness, support the successful implementation of the NLE, and ease the transition from nursing education to practice.

Consequently, considering training based on MOC within the context of the Egyptian NLE may offer substantive insights about the preparation for the examination, reducing the perception of stress, and assisting the effective practice of the newly introduced national licensure. Thus, this study aims to assess the impact of MOC-based training on the perceived stress of senior nursing students preparing for the Egyptian National Licensure Examination. It was expected that the MOC-based training would lead participants to report a greater post-intervention decrease in perceived stress than the control participants.

## 2. Research hypothesis

### 2.1. H_0_ (null hypothesis)

There is no statistically significant difference in perceived stress levels among nursing students transitioning to the new pre-licensure exam in Egypt before and after receiving MOC training.

### 2.1. H_1_ (alternative hypothesis)

Nursing students in Egypt who receive MOC training will exhibit a statistically significant reduction in perceived stress levels as they transition to the new pre-licensure exam compared to their stress levels before the training.

## 3. Methods

### 3.1. Research design, sample, and sampling

#### 3.1.1. Research design

A quasi-experimental comparative design approach was utilized in evaluating the effect of MOC training on perceived stress levels of final-year nursing students in Egypt who were preparing for the newly introduced pre-licensure examination. The study was conducted on 2 groups: one study group that was given structured MOC training sessions and one control group that continued with the standard curriculum. This design enabled assessment of stress changes in the 2 groups and individual participants pre and post intervention. Students were chosen with a non-probability purposive sampling method, which ensured that only students who met the eligibility criteria were included. This method allowed for an optimal and systematic evaluation of the effect of MOC training on stress during a clinically essential educational period.

#### 3.1.2. Setting

The research included nursing students from every part of Egypt to have a more balanced scope and variability in the study sample. It integrated 8 universities selected through stratified random sampling within the 4 main regions of the country such that one was from Northern Egypt, 2 from Upper Egypt, 2 from Eastern Egypt, 2 from Western Egypt, and one from the Cairo and Delta region. Including rural and urban campuses ensured balance within the entire population of nursing students in the country. Classrooms, computer labs, and clinical training areas were the study settings. Data collection and MOC training sessions were scheduled in collaboration with faculty members and the academic calendars.

#### 3.1.3. Study population

The research focused on the last-year undergraduate nursing students from certain nursing faculties in Egyptian universities because they were eligible to sit for the national pre-licensure examination for that academic year. The total sample was 1000 students, of which 500 students formed the study group and were given MOC training, while the other 500 formed the control group and received no extra training other than the standard curriculum.

Participants were recruited through nonprobability purposive sampling based on certain inclusion criteria such as being a final-year student, eligible for the licensure exam, and willing to provide informed consent. The selection of the universities was done through stratified random sampling, which aimed to ensure coverage from important areas of Egypt, such as Northern, Upper, Eastern, Western, and Central regions (Cairo and Delta). This approach was used to enable greater diversity and representativeness in the study population.

#### 3.1.4. Inclusion and exclusion criteria

This study’s inclusion criteria focused on final-year undergraduate nursing students from accredited nursing schools in Egypt who were eligible and scheduled to take the national pre-licensure exam in the current academic year. Participants had to provide informed consent and were required to complete both pre- and post-intervention evaluations. Students were excluded if they had a diagnosed psychological disorder, were receiving treatment for a mental health condition, were failed candidates or repeat candidates for the licensure exam, had participated in formal training or preparatory courses for the licensure exam outside the study’s intervention, or were unwilling or unable to complete the study’s data collection instruments in full.

#### 3.1.5. Sample size

The sample size was determined based on the primary outcome of the study, which was the difference in perceived stress scores measured by the Perceived Stress Scale (PSS-10) before and after the intervention. Assuming a moderate intervention effect size, a two-sided significance level of 0.05, and a statistical power of 80%, the required sample size was calculated using a comparative pre-post intervention design. Considering potential attrition and incomplete responses, the sample size was increased to achieve adequate statistical power. The final recruited sample consisted of 1000 students (500 participants in each group), which also provided sufficient power for multivariable regression analysis examining predictors of post-intervention perceived stress.

#### 3.1.6. Sampling procedure

A two-stage multistage sampling design was used in Egypt to enhance geographic diversity and capture variation related to the different educational environments. The first stage involved the application of a stratified random sampling technique to choose 8 nursing faculties from the primary geographic divisions of Egypt (Northern Egypt, Upper Egypt, Eastern Egypt, Western Egypt, and the Cairo/Delta region). The strategy of choosing institutions was designed to capture a range of geographic and educational contexts. However, it did not aim to attain comprehensive national representativeness of Egypt’s nursing students.

In the second stage, the final-year nursing students from the selected nursing faculties were sampled in accordance with the exposure and inclusion criteria for the study. This was done through the purposive sampling technique. Interested students were provided with a participant information sheet and had the opportunity to ask any questions. Those who met the criteria and provided informed consent were sampled for the study. The remaining students were then divided into 2 groups: the intervention group (n = 500), who received the structured MOC training program, and the control group (n = 500), who continued with the standard curriculum.

This sampling approach permitted capturing nursing students from a range of divergent instructional and regional contexts in accordance with the aim of the study. However, the findings of the study must be considered in light of the sampling approach and the representativeness of the study sample with respect to the entire population of final-year nursing students in Egypt.

#### 3.1.7. Data collection procedures

Data collection occurred over a three-month timeframe spanning from December 2024 to March 2025. Following the receipt of ethical approval and administrative clearance from the collaborating universities, eligible final-year nursing students were located and engaged through on-campus and official university email notifications. Written informed consent was obtained from participants meeting the criteria, enrolling them in the study. During the baseline phase, all participants, regardless of study group assignment, completed a demographic survey and the PSS-10. Within the baseline phase, the study group was granted an MOC training intervention consisting of 4 weeks of intensive training, while the control group reverted back to their normal academic routines. Following the completion of the training and shortly before the pre-licensure examination, both groups were re-assessed using the PSS-10 to measure post-intervention stress levels. Surveys were administered online and in person during sessions lasting 15 to 20 minutes. Responses were stored confidentially and processed without identifiers to ensure anonymity.

## 4. Data collection tools

### 4.1. Perceived stress scale (PSS)

Part I: Demographic data will be used to gather baseline data including age, gender, academic GPA, study hours per day, and prior test-taking experience.

Part II: The PSS-10, developed by^[27]^, was used to assess students’ perceived stress levels. It measures 2 dimensions: Perceived Helplessness, reflecting feelings of being overwhelmed and out of control, and Perceived Self-Efficacy, capturing confidence in managing stressors. The scale consists of 10 items rated on a 5-point Likert scale ranging from 0 (never) to 4 (very often). The total score ranges from 0 to 40, with higher scores indicating higher perceived stress. The PSS has been validated in various populations, including university students,^[28–30]^ and shows strong internal consistency (Cronbach alpha > 0.80). Scoring system 0 to 13: Low stress, 14 to 26: Moderate stress, 27 to 40: High stress. Items 4, 5, 7, and 8 are reverse scored.

## 5. Structured MOC training sessions

The MOC training program is a newly developed, standardized, and structured program designed to assist final-year nursing students with the Egyptian national pre-licensure examination. The program intends to ease examination-induced stress, strengthen basic nursing knowledge and clinical reasoning skills, and improve self-efficacy and students’ confidence and preparedness for the licensing exam.

The intervention took place over 4 consecutive weeks, consisting of 4 training days each week (16 sessions total). Each training day consisted of structured educational components that integrated theory, application, assessment, and feedback, along with stress management techniques. The program consisted of both in-person and, as deemed necessary, online sessions. The same content, learning goals, and structure of a session were maintained for all delivery formats.

The training covered content and skills related to the major competency areas of the national nursing pre-licensure examination. Each week, the focus was on a particular discipline of nursing: Critical and Medical-Surgical Nursing, Obstetric and Midwifery Nursing, Pediatric Nursing, and Nursing Administration and Leadership. The teaching was done by nursing faculty members with a focus on that particular discipline. All trainers had prior experience in the field of nursing as well as teaching and competency-based assessments, and were given a formalized training session to ensure that all trainers were on the same page with regard to teaching methods, learning objectives, and session outlines.

To ensure consistency across intervention delivery, trainers were given access to a uniform training manual. To ensure each trainer implemented the intervention as intended, researchers conducted active site visits, checked session completion logs, and reviewed trainer notes. Session logs were evaluated to monitor attendance, and the amount of exposure participants received to the intervention was assessed weekly during the four-week training.

For the first 2 training days of each week, theory-based training was structured and constructed with the aid of standardized lectures. These lectures included visual aids, clinical examples, and case presentations. Summaries of the lectures were provided to students, and multiple interactive components were utilized, including quizzes and problem- posing and-solving exercises, to assist in active learning.

To further assist in the application of theory, training during the last 2 days of each week was dedicated to the national licensing exam preparation. This included a simulated exam of the actual national licensing exam. Small group discussions were used after each of the practice and simulated tests to enhance the participants’ clinical judgment, and decision-making and improve their ability to apply clinical skills. Evaluation of test performance, and bridging of the gaps in participants’ knowledge was accomplished through the provision of personalized feedback and recommendations.

Incorporating emotional coping strategies across the intervention aimed to improve participants’ psychological coping and readiness to sit an exam. This included guided breathing, mindfulness, and coping exercises, along with time management. Learning and emotional support were fostered through collaboration during group discussions.

As a measure to restrict contamination across participants, all intervention resources, materials, mock exams, and training resources were kept exclusive to participants of the intervention group for the duration of the study. Participants in the control group carried on with their normal academic activities and were not provided access to the MOC sessions and materials until the post-intervention assessment was completed.

At the end of the fourth week, there was an exhaustive review session that covered all of the topics that had been taught so far. Participants were required to complete a full-length cumulative mock exam that was administered under the same conditions as the national licensing examination. This was succeeded by a group debrief and an individualized feedback session. In its totality, the intervention offered a consistent blend of examination preparation, knowledge reinforcement, and stress management. This strengthened the intervention participants’ academic and psychological preparedness for the national pre-licensure examination.

## 6. Data analysis

Data were analyzed using IBM SPSS Statistics software, Version 26. Descriptive statistics were used to summarize participants’ demographic and academic characteristics, including frequencies, percentages, means, and standard deviations. Baseline characteristics between the intervention and control groups were compared using the chi-square test for categorical variables and independent samples *t*-tests for continuous variables to assess group comparability before the intervention.

The perceived stress scores were compared between the intervention and control groups at pre- and post-intervention assessments using independent samples t-tests. Within-group changes in perceived stress scores from pre- to post-intervention were examined using paired samples *t*-tests. One-way analysis of variance (ANOVA) was used to assess differences in perceived stress scores according to levels of computer skills competency.

Because a statistically significant baseline difference was identified between groups regarding family size, this variable was considered a potential confounding factor and was included as a covariate in the multiple linear regression analysis. Multiple linear regression was performed to identify predictors of post-intervention perceived stress scores, including age, gender, academic GPA, computer skills competency, smoking status, number of family members, and awareness of the pre-licensure examination.

The assumptions of parametric tests were assessed before analysis. Normality of residuals for regression analysis was evaluated using the Shapiro–Wilk test and visual inspection of Q–Q plots, which indicated an acceptable normal distribution (*P* > .05). Skewness and kurtosis values for the main study variables were within the acceptable range (−1 to +1), supporting approximate normality. Homogeneity of variances was assessed using Levene test, which demonstrated non-significant results (*P* > .05), confirming the assumption of equal variances.

For the regression model, multicollinearity among regression predictors was assessed using the variance inflation factor (VIF). VIF values ranged from 1.00 to 2.90, with the highest values observed for awareness of the pre-licensure examination (VIF = 2.90) and gender (VIF = 2.70). These values were below the accepted threshold of 5, indicating that no serious multicollinearity problems were present. Therefore, the regression coefficients were considered sufficiently stable for interpretation. The regression model was also evaluated for compliance with normality and homoscedasticity assumptions. Statistical significance was set at a *P*-value < .05 for all analyses.

## 7. Results

As presented in Table 1, the mean age of the participants in the study group (n = 500) and the participants in the control group (n = 500) showed no significant differences (60% vs 62% under 22 years, *P* > .05), suggesting that participants were similarly distributed across age categories. As with age, the gender composition in both groups was statistically equivalent; the study group contained 26% males, while the control group comprised 30.8% males (*P* > .05), suggesting gender was equally represented. Also, regarding academic performance, both groups exhibited comparable distributions in class GPA (36% vs 38.8% with GPA < 3, *P* > .05), suggesting comparable academic performance. Participants’ computer skills demonstrated no significant difference in competency levels (Good: 44% in study group vs 37.6% in control group, *P* > .05), suggesting that both groups had comparable levels of digital literacy. In addition, the groups were comparable regarding smoking and smoking information (14% vs 18% smokers, *P* > .05; 47.4% vs 53.2% smokers, *P* > .05), supporting baseline equivalence in knowledge exposure. A significant difference was observed between groups regarding family size (*P* < .05); therefore, family size was considered a potential confounding variable and was adjusted for in the regression analysis.

**Table 1:** Comparison of baseline demographic characteristics between study and control groups (n = 1000).

Variable	Study group (n = 500)	Control group (n = 500)	Total (n = 1000)	χ^2^ *P* value
	N (%)	N (%)	N (%)	
Age				
<22	300 (60%)	310 (62%)	610 (61.3%)	>.05
≥22	200 (40%)	190 (38%)	390 (38.7%)	
Gender				
Male	130 (26%)	154 (30.8%)	284 (28.4%)	>.05
Female	370 (74%)	346 (69.2%)	716 (71.6%)	
Academic GPA (last semester)				
<3	180 (36%)	194 (38.8%)	374 (37.4%)	>.05
≥3	320 (64%)	306 (61.2%)	626 (62.6%)	
Computer skills competency level				
Poor	80 (16%)	95 (19%)	175 (17.5%)	>.05
Moderate	200 (40%)	217 (43.4%)	417 (41.7%)	
Good	220 (44%)	188 (37.6%)	408 (40.8%)	
Smoking				
Smoker	70 (14%)	90 (18%)	160 (16.0%)	>.05
Nonsmoker	430 (86%)	410 (82%)	840 (84.0%)	
Number of family members				
<5	300 (60%)	153 (30.6%)	453 (45.3%)	<.05
≥5	200 (40%)	347 (69.4%)	547 (54.7%)	
Having information about pre-licensure exam				
Yes	237 (47.4%)	266 (53.2%)	503 (50.3%)	>.05
No	263 (52.6%)	234 (46.8%)	497 (49.7%)	

Table 2 demonstrates the comparison of perceived stress scores according to demographic and academic characteristics before and after the intervention. Before the intervention, no significant differences were observed between the intervention and control groups across the examined characteristics, indicating baseline comparability. Following the intervention, the intervention group showed significantly lower perceived stress scores compared with the control group across most subgroups. Additionally, within the intervention group, lower post-intervention stress levels were observed among students with higher academic GPAs, better computer skills, and prior knowledge of the pre-licensure examination. Detailed statistical comparisons are presented in Table 2.

**Table 2:** Comparison of pre- and post-test perceived stress scores by participant characteristics in study and control groups.

Variable	Nursing students perceived stress (mean ± SD)					*t* test *P* value
	Pretest		*t* test *P* value	Post-test		
	Study group N = 500	Control group N = 500		Study group N = 500	Control group N = 500	
Age						
<22	28.9 ± 5.6	29.3 ± 6.1	>.05	12.0 ± 3.1	31.6 ± 7.2	<.05
≥22	28.6 ± 5.8	29.0 ± 5.9	>.05	12.2 ± 3.3	31.4 ± 7.0	<.05
*P*-value	>.05	>.05		>.05	>.05	
Gender						
Male	28.5 ± 5.9	29.0 ± 6.3	>.05	12.3 ± 3.4	31.2 ± 7.3	<.05
Female	29.0 ± 5.6	29.3 ± 5.9	>.05	12.0 ± 3.1	31.6 ± 7.0	<.05
*P*-value	>.05	>.05		>.05	>.05	
Academic GPA (last semester)						
<3	30.2 ± 5.4	31.0 ± 6.2	>.05	13.2 ± 3.5	32.7 ± 6.9	<.05
≥3	27.1 ± 5.6	27.8 ± 5.7	>.05	11.0 ± 2.8	30.0 ± 7.2	<.05
*P*-value	<.05	<.05		<.05	<.05	
Computer skills competency level						
Poor	30.1 ± 5.5	30.7 ± 6.4	>.05	13.1 ± 3.4	32.3 ± 7.1	<.05
Moderate	28.6 ± 5.7	29.1 ± 5.9	>.05	12.0 ± 3.1	31.3 ± 6.9	<.05
Good	27.2 ± 5.6	28.1 ± 5.7	>.05	11.0 ± 2.9	30.0 ± 7.0	<.05
ANOVA test *P*-value	<.05	<.05		<.05	>.05	
Smoking						
Smoker	30.0 ± 5.6	30.9 ± 6.2	>.05	13.4 ± 3.5	32.8 ± 7.1	<.05
Nonsmoker	28.1 ± 5.6	28.9 ± 5.8	>.05	11.7 ± 3.0	30.5 ± 6.8	<.05
*P*-value	<.05	<.05		<.05	<.05	
Number of family members						
<5	28.6 ± 5.6	29.0 ± 5.8	>.05	11.9 ± 3.1	30.7 ± 7.0	<.05
≥5	29.1 ± 4.8	29.6 ± 7.1	>.05	12.4 ± 3.3	32.1 ± 7.2	<.05
*P*-value	>.05	>.05		>.05	>.05	
Having information about pre-licensure exam						
Yes	27.6 ± 5.5	28.5 ± 5.2	<.05	11.3 ± 3.0	30.2 ± 6.8	<.05
No	30.0 ± 5.8	30.5 ± 6.1	>.05	13.1 ± 3.4	33.1 ± 7.3	<.05
*P*-value	<.05	<.05		<.05	<.05	

ANOVA = analysis of variance.

Table 3. reported that there was no significant difference between the study group (M = 28.8, SD = 5.7) and the control group (M = 29.2, SD = 6.0) in pre-intervention; *t* = 1.08, *P* > .05. At post-test, however, the study group reported significantly lower stress scores (M = 12.1, SD = 3.2) compared to the control group (M = 31.1, SD = 7.1); *t* = 55.70, *P* < .001. Also, there was a significant reduction in stress scores from pretest to post-test; *t* = 57.71, *P* < .001. In contrast, the control group exhibited a significant increase in stress scores between pre- and post-tests; *t* = 5.12, *P* < .001.

**Table 3:** Within-group comparison of pre- and post-test stress scores.

Group	Time	Mean ± SD	Interpretation	*t*-test	*P*-value	Cohen effect size
Study group	Pre	28.8 ± 5.7	High	57.71	<.05	
	Post	12.1 ± 3.2	Low			2.58
Control group	Pre	29.2 ± 6.0	High	5.12	<.05	
	Post	31.1 ± 7.1	High			0.23
Study vs control	Study pre	28.8 ± 5.7	High	1.08	>.05	
	Control pre	29.2 ± 6.0	High			0.07
Study vs control	Study post	12.1 ± 3.2	Low	55.70	<.05	
	Control post	31.1 ± 7.1	High			3.45

A multiple linear regression analysis was performed to study the factors predicting perceived stress scores after the test among nursing students (*N* = 1000). The overall model was significant with *F* = 194.6, *P* < .001, *R*^2^ = .540, suggesting that 54% of the variance in perceived stress was explained by the factors. The effect size was large (*f*^2^ = 1.17), indicating that the findings had considerable practical relevance. As for the predictors, prior academic GPA, computer skills competency, and having information regarding the pre-licensure exam were all associated with significantly lower perceived stress, indicating a negative effect (β = −0.18, *P* < .001; β = −0.17, *P* < .001; β = −0.18, *P* < .001). Smoking status, however, showed a positive effect predicting higher stress among smokers (β = 0.15, *P* < .001). Family size was not significantly associated with post-intervention perceived stress scores (β = −0.04, *P* = .266), indicating that the baseline difference in family size did not significantly contribute to differences in perceived stress after adjustment. Diagnostic evaluation of the regression model indicated acceptable levels of multicollinearity. VIF values ranged from 1.00 to 2.90, with the highest values observed for awareness of the pre-licensure examination (VIF = 2.90) and gender (VIF = 2.70). Although these variables showed relatively higher collinearity compared with other predictors, the values remained within acceptable limits, suggesting minimal residual multicollinearity effects and supporting the reliability of the regression estimates. Table 4.

**Table 4:** Multiple linear regression predicting post-test perceived stress score among nursing students (N = 1000).

Variables	*Β* coefficient	SE	Beta	*T*	*P*-value	VIF
Age	−0.32	0.28	−0.03	−1.14	.253	1.56
Gender (1 = male, 0 = female)	−0.75	0.43	−0.04	−1.74	.082	2.7
Academic GPA (1= ≥3)	−2.61	0.36	−0.18	−7.25	<.05	1.00
Computer skills (1 = good)	−3.42	0.45	−0.17	−7.60	<.05	1.90
Smoking (1 = smoker, 0 = nonsmoker)	2.73	0.38	0.15	7.18	<.05	1.07
Number of family members	−0.36	0.27	−0.04	−1.12	.266	1.5
Information about pre-licensure exam (1 = yes, 0 = no)	−4.87	0.40	−0.18	−8.05	<.05	2.9

Family size was included in the regression model because of the significant baseline difference between intervention and control groups. VIF values ranged from 1.00 to 2.90. The highest VIF values were observed for awareness of the pre-licensure examination (2.90) and gender (2.70), remaining below the threshold indicating problematic multicollinearity.

*R* =0.735, *R*^2^ = 0.540, adjusted *R*^2^ = 0.536, *F* = 194.6, *P* < .05, effect size =1.17.

VIF = variance inflation factor.

## 8. Discussion

This study offers preliminary data indicating that Egyptian nursing educators may use MOC training sessions as an effective “stress-reduction” tool to ease nursing students’ anxiety regarding the introduction of the new pre-licensure examination. Results suggest that the focus of examination-related stress may extend beyond the examination itself, encompassing students’ personal characteristics, preparedness, and confidence. Since MOC training sessions offer structure, a system, and an understanding of the examination, they may help provide the necessary control and reduce students’ stress-related uncertainty about the examination. Additionally, the results indicate differences in adaptation to new examination-related stress among students, and the demographic differences may be related to students’ academic characteristics.

This interpretation corroborates previous studies whereby students’ adaptation to high-stakes assessments and challenges is substantially influenced by demographic and psychosocial factors. For instance, a randomized controlled trial among Turkish nursing students, with a mean age of 19.97 ± 1.39 years, reported a significant difference between nursing students’ age and level of stress.^[31]^ The results show that age, which could mean maturity, life responsibilities, or academic progress, can influence stress levels. The results show that the study group had much lower stress levels after the intervention, while the control group had higher stress levels. The findings indicate that the degree of perception and handling of stress among students is influenced by differences related to age, maturity level, academic level, and even life experiences.

Furthermore, the present study’s findings revealed that there was a statistically significant difference between nursing students’ genders in their perception of stress. This finding echoes findings of literature in Egypt,^[32]^ in Saudi Arabia.^[33,34]^ This suggests that gender substantially affects the experience and interpretation of stress within the realm of nursing education. However, these findings must be interpreted cautiously because gender variation in stress response may be influenced by sociocultural and educational contexts and may not generalize globally. Differences in gender roles, coping strategies, perceived expectations, and access to support services may contribute to differences across studies. These findings highlight the imperative for gender-sensitive approaches in the formulation of stress management interventions and support services within nursing education programs, guaranteeing that the distinct requirements of all students are sufficiently addressed.

The present study’s findings revealed that the students who had information about the pre-licensure exam and computer skills competency had significantly lower stress scores. A study discussing the level of preparedness and confidence of graduating students for the Licensure Examination reported that students with computer skills competency and who were well prepared had significantly lower stress levels.^[35]^ Therefore, MOC training appears to improve familiarity and control, particularly for students during transitional periods of new assessment systems. The impact will depend on student attributes, the level of institutional support, and whether the primary source of stress is the examination or general academic and clinical stress.^[36]^

In contrast, the findings of the current study oppose those of Ali et al (2022), who investigated perceived stress among nursing students in Egypt and demonstrated a statistically negligible connection between the PSS and the students’ socio-demographic factors.^[37]^ There are several reasons for this gap. The current study was conducted when a new national pre-licensure examination system was being introduced in the country. This system came with new demands, uncertainties, and procedures related to examinations and preparation. Other studies may have been conducted in traditional testing environments and procedures and may have focused on other dimensions of student-related stress. The differences in findings could be related to methodologies, including but not limited to sample selection, measurement methods, and data collection schedules. These disparities highlight the necessity of accounting for environmental and methodological variables when interpreting findings associated with psychological categories like subjective stress.

The regression analysis further underscored this variability, revealing that prior academic GPA, computer skills competency, having information regarding the pre-licensure exam, and smoking status collectively accounted for 54% of the variance in perceived stress. This proportion indicates that these factors accounted for a considerable amount of variability in perceived stress in the current sample. Still, other psychological, social, and institutional factors that are not measured may also explain the stress students experience. Students exhibiting superior academic success presumably had heightened confidence in their abilities, while those possessing advanced computer skills showed more ease in managing the digital components of the examination. Furthermore, students with prior knowledge of the pre-licensure exam style and requirements likely encountered diminished uncertainty, a recognized factor in academic stress.

These findings align with the current literature emphasizing the significance of academic readiness and technological proficiency in alleviating exam-related anxiety. Comparable research in several educational settings indicates that early familiarity with exam forms and organized, prepared activities can substantially alleviate stress and enhance results.^[38,39]^ Early exposure to examinations for nursing students is a vital aspect of their education, as it can markedly affect their academic performance, retention rates, and professional skill acquisition. The amalgamation of early clinical exposure and problem-based learning has demonstrated an enhancement in nursing students’ educational experiences by merging theoretical knowledge with practical application. This method enhances their academic self-efficacy and equips them for practical clinical environments.

Within-group comparison of pre- and post-test stress scores showed a significant reduction in stress scores from pretest to post-test in the study group. In contrast, the control group exhibited a significant increase in stress scores between pre- and post-tests. The significant effect of MOC training sessions on perceived stress levels among nursing students can be attributed to the structured approach these sessions provide in managing stress and enhancing coping mechanisms. However, the study suggests that reduced perceived stress is associated with MOC training, but it doesn’t provide evidence that MOC training leads to long-term stress reduction. Nursing students often face high levels of stress due to the demanding nature of clinical training, which can impact their mental health and academic performance. MOC training sessions, which are designed to improve clinical competence and reduce stress, have shown promising results in alleviating perceived stress among nursing students.

This result is corroborated by other studies that emphasize the efficacy of organized training programs in alleviating stress and enhancing coping skills among nursing students. A study in Türkiye indicated that stress management programs tailored for nursing students significantly reduced felt stress levels, as reflected in alterations in the felt stress scale scores.^[40]^ A separate investigation into the impact of portfolio-based education and assessment on the clinical competence of nursing students at Ahvaz Jundishapur University of Medical Sciences indicated no significant difference between the 2 educational cohorts in the pretest. However, notable differences were observed in the average scores of clinical competence across all 3 domains before and after the implementation of the portfolio-based education method.^[41]^ Furthermore, a comprehensive review and meta-analysis evaluated the efficacy of stress management interventions for nursing students, indicating that such interventions considerably alleviated psychological stress, with more pronounced effects noted in on-site programs. These therapies resulted in notable reductions in physiological stress indicators, including blood pressure, heart rate, and cortisol levels.^[42]^ The different outcomes from the mentioned interventions suggest that stress management programs should reflect students’ individual needs and be a consistent part of educational and psychological support programs.

The findings advocate for the integration of MOC training as a standard component of nursing curricula, especially during the implementation of new assessment systems. Institutions ought to implement measures that improve students’ digital literacy and provide explicit, prompt information on examination requirements. These measures could alleviate stress and foster fairer examination experiences among varied student demographics.

While MOC training was likely to lead to a substantial reduction in perceived stress, the large effect size was likely an artifact of methodological considerations and should be interpreted with caution. It cannot be ruled out that the stress reduction represented positive preparedness and familiarity that would transfer to the students as the new pre-licensure examination is introduced. Self-reported stress was subject to social desirability bias and demand characteristics, and students may have wished to report a positive outcome, given their awareness of the improving intervention. Additionally, the lack of concealment of the allocation would likely have introduced expectancy effects. Therefore, while MOC training would likely improve perceived stress and would likely be useful, further randomized controlled trials to determine the effect size and duration would be warranted to ascertain the effect this training would have on participants’ stress levels.

## 9. Limitations

The current investigation notes the potential of MOC training as a support system for nursing students and for adaptation to a new high-stakes testing system. Some limitations have to be addressed. First, the combination of methods to assess stress was missing. Stress was assessed only using the self-reported PSS-10. Like other surveys, this has the potential for both recall bias and socially desirable responding. Objective and other methods to assess stress are needed for the following investigations. The second limitation was the absence of extended follow-up, which left unexamined the short- and long-term effects of reduced stress on (real) performance in the (real) pre-licensure exam. Future studies should assess participants’ stress in the lead-up to the licensure exam and on the day of the exam. The third limitation was the absence of random allocation, expectancy effects, and sample contamination. These limitations, in combination with the missing assessment of a number of key factors, including academic workload, personal stressors, support from faculty, and characteristics of the institution, severely limit the interpretation of the results. Finally, given the study’s specific context, the results should be applied cautiously.

## 10. Conclusion and implications for nursing practice

The aim of this study was to investigate the effect of MOC training on the level of stress of nursing students regarding the upcoming nursing pre-licensure exam in Egypt. In Egypt, MOC training involves reviewing the clinical content, planning your approach to the exam, and practicing stress management techniques. It gets students mentally and emotionally ready for this career-changing moment in their lives. Students who took MOC training reported lower stress levels. The preparation was statistically stress-relieving for the students who were about to take the exam. Education systems in nursing that prepare students only to take the professional licensure exams have the responsibility to revise their curricula to meet the psychological and emotional needs of students. This is a clear sign of the need among nursing faculties to incorporate systems combining various review methodologies, practice, and supportive psychology into the final year of pre-licensure nursing exam preparation. The Egyptian Health Council recommends that cooperation with nursing education institutions can contribute to the development of comprehensive pathways for nursing licensure, with supportive psychological support to help with the emotional and psychological preparedness of nursing graduates and a fair transition to professional nursing practice. MOC training will be extended beyond confidence and clinical competence to include examination, licensure, and professional and clinical engagement metrics.

## Acknowledgments

The authors express their gratitude to the Princess Nourah bint Abdulrahman University Researchers Supporting Project number (PNURSP2026R312), Princess Nourah bint Abdulrahman University, Riyadh, Saudi Arabia.

## Author contributions

**Conceptualization:** Hasan Ahmed Awad Basyouny, Mohamed Hashem Kotp, Hossam Ali Ismail, Ahmed Khalaf Mekdad.

**Data curation:** Hasan Ahmed Awad Basyouny, Mohamed Hashem Kotp, Hossam Ali Ismail, Ahmed Khalaf Mekdad, Ahmed Ali Hafez, Shimaa Ramadan Ahmed.

**Formal analysis:** Ahmed Shaaban Attia, Hasan Ahmed Awad Basyouny, Mohamed Hashem Kotp.

**Funding acquisition:** Wafa Hamad Almegewly.

**Investigation:** Abdelaziz Hendy, Rasha Kadri Ibrahim.

**Methodology:** Rasha Kadri Ibrahim, Rasha Mohammed Hussien.

**Resources:** Ahmed Shaaban Attia, Ahmed Ali Hafez.

**Software:** Abdelaziz Hendy.

**Validation:** Ahmed Shaaban Attia, Eman Atef Ali.

**Visualization:** Ahmed Khalaf Mekdad, Shimaa Ramadan Ahmed.

**Writing – original draft:** Hasan Ahmed Awad Basyouny, Mohamed Hashem Kotp, Abdelaziz Hendy, Rasha Kadri Ibrahim, Hossam Ali Ismail, Ahmed Shaaban Attia, Ahmed Khalaf Mekdad, Sameer A. Alkubati, Mohamed Ahmed Aly, Eman Atef Ali, Shimaa Ramadan Ahmed.

**Writing – review & editing:** Abdelaziz Hendy, Rasha Kadri Ibrahim, Hossam Ali Ismail, Ahmed Ali Hafez, Eman Atef Ali, Rasha Mohammed Hussien, Shimaa Ramadan Ahmed, Wafa Hamad Almegewly, Sameer A. Alkubati, Mohamed Ahmed Aly.
